# Quantitative Bone Scintigraphic Follow-Up After a 2-Month Cross-Training Program Including Swimming in Showjumping Horses with Back and Neck Pain

**DOI:** 10.3390/ani16162526

**Published:** 2026-08-13

**Authors:** Antoine Prémont, Claire Moiroud, Sandrine Jacquet, Audrey Beaumont, Lélia Bertoni, Henry Chateau, Fabrice Audigié

**Affiliations:** 1 ACAP3, Ecole Nationale Vétérinaire d’Alfort, F-14430 Goustranville, France; claire.moiroud@vet-alfort.fr (C.M.); sandrine.jacquet@vet-alfort.fr (S.J.); audrey.beaumont@vet-alfort.fr (A.B.); lelia.bertoni@vet-alfort.fr (L.B.); henry.chateau@vet-alfort.fr (H.C.); fabrice.audigie@vet-alfort.fr (F.A.); 2 Ecole Nationale Vétérinaire d’Alfort, F-94700 Maisons-Alfort, France

**Keywords:** swimming, scintigraphy, back, neck, horse

## Abstract

To quantitatively describe changes in skeletal radiopharmaceutical uptake during a training program including swimming in horses with neck and back pain, eighteen showjumping horses with documented vertebral lesions were prospectively included in a training program composed of 4 weeks of ridden work and then 8 weeks combining swimming and ridden exercise. Previously published data on this cohort did not demonstrate any clear change in thoracolumbar mobility. Bone scintigraphy was performed at the 4th and 12th weeks. Radiopharmaceutical uptake was quantified in 205 regions of interest (ROIs) and normalized using a Z-score approach. For each ROI, a linear mixed-effects model was fitted to evaluate the effect of the time point (W4, W12) on the normalized radiopharmaceutical uptake with the horse as a random effect. Overall variation in normalized signal intensity (Z-score difference W12–W04) was small (mean < 0.001; range −0.28 to 0.24). Significant changes between time points were identified in 21 ROIs. Thirteen ROIs located in the axial skeleton showed a decreased radiopharmaceutical uptake, whereas eight ROIs located in the limbs, including the humeral tubercles on both lateral views, showed an increased uptake. During a 2-month training program including swimming, axial skeletal radiopharmaceutical uptake did not increase in this cohort of horses with neck and back pain. However, substantial interindividual variability in scintigraphic changes was observed. Further controlled studies are needed to confirm these preliminary results and to better explore the effects of swimming on horses with axial musculoskeletal disorders.

## Simple Summary

Swimming is frequently used in the training and rehabilitation of sport horses, but little evidence exists regarding its effects on horses with neck and back pain. Because swimming may place the spine in an extended position, it has traditionally been considered unsuitable for horses with back or neck lesions. This study investigated changes in bone metabolism during a 12-week training program that included 8 weeks of swimming in 18 showjumping horses affected by neck and/or back pain. Bone scintigraphy was performed before and after the swimming phase, and radiopharmaceutical uptake was quantitatively measured in 205 skeletal regions. Overall, scintigraphic changes were small. Most significant changes in the spine corresponded to a decrease in radiopharmaceutical uptake, suggesting no increase in bone stress or inflammation in the axial skeleton during the program. In contrast, some regions of the limbs, particularly the humeral tubercles, showed increased uptake, potentially reflecting adaptation to the muscular demands of swimming. These findings combined with the previous results on the same horses showing stable thoracolumbar mobility through the program suggest that a training program including swimming was generally well tolerated by showjumping horses with neck and back pain under the conditions of this study. Further controlled studies are needed to better understand the effects of swimming on horses with axial musculoskeletal disorders.

## 1. Introduction

Swimming is often used for exercise training of equine athletes [1] or as a rehabilitation modality [2,3]. A few studies have investigated horses’ physiological response to swimming [4,5,6,7,8] and underwater biomechanics [9,10,11,12,13], but clinical studies are rare, and the rationale for its use is mostly based on empirical data [14,15]. The relationship between swimming and the development or worsening of several medical conditions such as arrhythmias [16], exercise-induced pulmonary hemorrhage [17], upper airway collapse [18] and colic [19] has been explored. However, even though aquatic training is often used for rehabilitation of musculoskeletal injuries, the evidence is poor regarding indications or contraindications of swimming for locomotor injuries. The axial skeleton is a very common site of pain in sport horses, and axial lesions are often performance-limiting for showjumpers [20]. Those injuries can often be the reason for a horse to enter a rehabilitation program or be concomitant of other lesions that would benefit from rehabilitation modalities such as swimming. Because of the extended position of the spine underwater, back and neck injuries have been considered as a contraindication to swimming in horses [14,21]. Recent studies have, however, demonstrated that horses’ position and swimming kinematics are susceptible to large interindividual variations [9] and that many horses demonstrate only limited degree of extension of the back and low neck [11].

Bone scintigraphy is very sensitive to recent and active bone remodeling. It has been used to monitor the changes induced by training [22], or rehabilitation [23,24] and response to treatment [25]. It has a good sensitivity and specificity in identifying axial bone, joint and muscle lesions and is considered a good technique to assess the clinical relevance of radiographic findings [26,27,28,29,30,31]. The radiopharmaceutical uptake of a bone lesion changes quickly in response to changes in bone remodeling and inflammation, making scintigraphy a suitable modality for monitoring such lesions [23,24]. Axial skeletal pain is usually diagnosed by a combination of clinical evaluation and radiographic and ultrasonographic imaging [20], but axial lesions demonstrate slow radiographic changes, making radiography less relevant for short term follow-up.

Therefore, the purpose of this exploratory study was (1) to quantify the changes in skeletal metabolism using bone scintigraphy in showjumping horses with neck and back pain during a training program involving swimming and (2) to describe scintigraphic changes in the limbs of non-lame horses during a cross-training program including swimming. We hypothesized that no or little consistent scintigraphic changes would be observed after 2 months of cross-training but that the scintigraphic changes in response to this training would vary greatly between individuals.

## 2. Materials and Methods

### 2.1. Horses

Eighteen horses aged between 5 and 14 years (mean age ± standard deviation SD: 9.6 ± 2.8 years) were included in this prospective study. They consisted of 6 mares and 12 geldings. There were 15 Selle Français, 1 Zangersheide, 1 KWPN and 1 Poney Français de Selle. All horses were regularly trained in showjumping for at least a year before admission, and most of them were actively competing. A history of poor performance or work-related problems attributed to back or neck pain was reported for all horses. All horses performed at amateur level in showjumping (between 1m10 and 1m30) before their inclusion in the study, and all went back to their previous level of competition after the program. An initial standard locomotor evaluation was performed by an ACVSMR/ECVSMR diplomate (CM) including examination at the walk, trot, and canter. A standard radiographic examination of the axial skeleton (from C1 to L5) was performed as previously described [20,32]. Standard ultrasound examination of the caudal neck (C5-T1) and transrectal pelvic ultrasound examination were performed by an ACVSMR/ECVSMR diplomate (CM) as previously described [33,34,35,36]. Images were stored and reviewed after anonymization for lesion characterization by 3 sports medicine specialists (CM, SJG, and LB) and an ECVDI diplomate (FA). Investigators were blinded to the clinical investigation and the horse history. Inclusion criteria comprised radiographic evidence of cervical or thoracolumbar lesions and no treatment received in the last 2 months. Horses with isolated sacroiliac lesions without concurrent cervical or thoracolumbar involvement, limb-related locomotor disorders, or signs of ataxia or incoordination were not included. Final criteria for inclusion was a successful trial session in the swimming pool judging the ability of the horse to safely enter and exit the pool before entering the program. Six horses were excluded prior to starting the program because of lameness (2/6) or unwillingness to enter the swimming pool (4/6). One horse was excluded after partial completion of the program because of skin lesions and myositis. Multiple lesions of various severity were recorded in each horse. To help identify potential factors associated with scintigraphic changes, horses were classified in 2 clinical groups (5 in the cervical group, 13 in the thoracolumbar group) based on the localization of their predominant, most severe lesion as previously described [37]. The classification is reported in Table 1.

Additional data from some horses included in this study were used in previously published work [9,10,37].

### 2.2. Study Design

Prior to the procedure, the protocol was examined and approved by the dedicated clinical research ethics committee (Comité d’Ethique en Recherche Clinique, ENVA, N◦ 2022-09-19). After the inclusion examination, all horses were enrolled for 12 weeks in a standardized training program in the same rehabilitation center (KINESIA CIRALE—Locomotor Pathology Unit of the Equine Veterinary Teaching Hospital at the National Veterinary School of Alfort (EnvA)). The recruitment of horses was spread over a 30-month period from December 2022 to June 2025. Horses received a full clinical and imaging evaluation including a standard full body scintigraphy at the end of the 4th and 12th weeks of the protocol. This protocol was previously described [37] and consisted of a land training phase of 4 weeks followed by an 8-week cross-training phase, during which three land-based training sessions per week were replaced by swimming sessions of progressively increasing intensity. The program is summarized in Table 2. Throughout the entire program, each horse was assigned to be ridden by one of the 2 experienced riders of the center. To ensure the horses were sound and able to continue the program, they received weekly clinical follow-ups by an ACVSMR/ECVSMR diplomate (CM).

### 2.3. Scintigraphy

**Figure A1:**
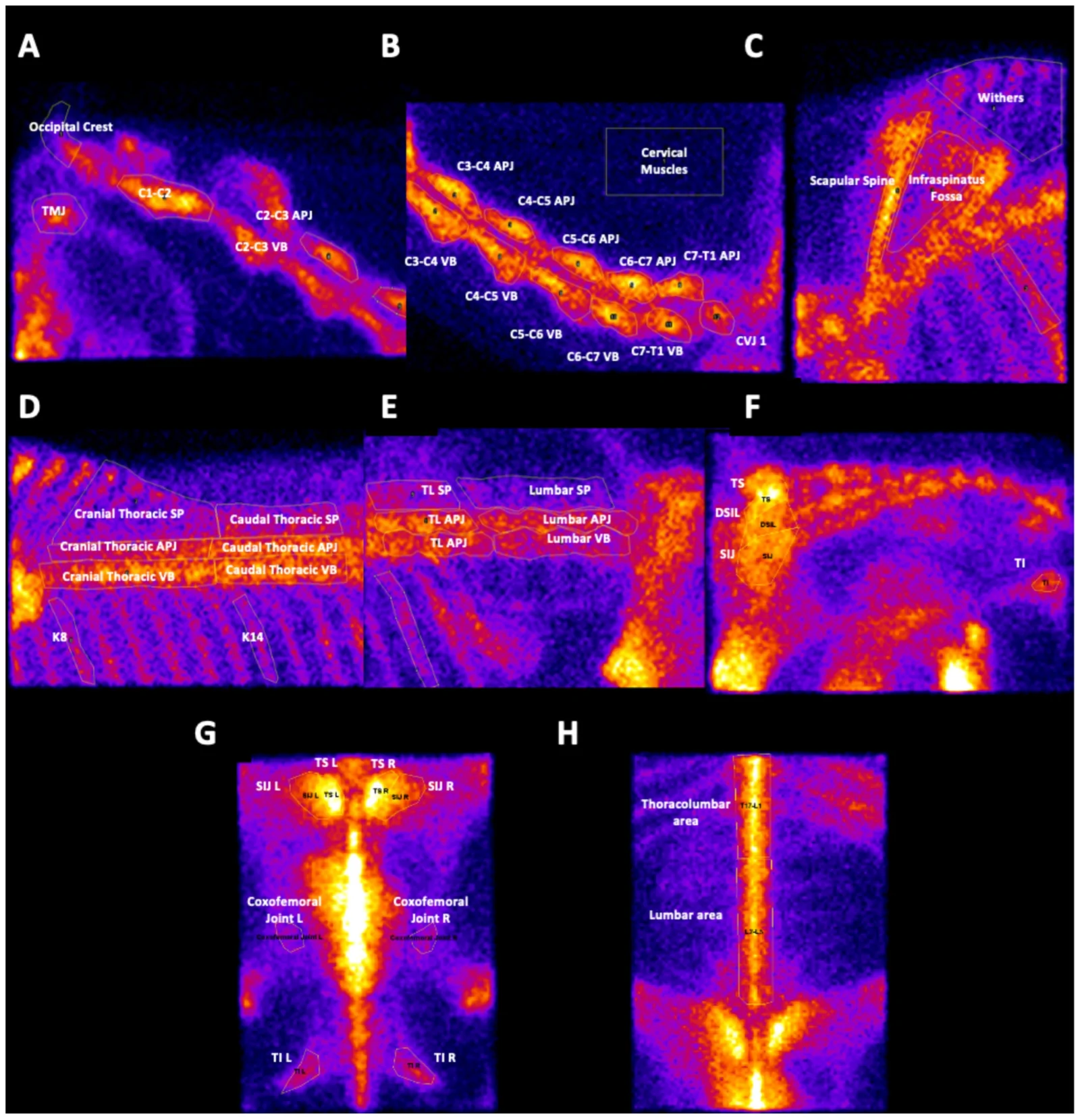
Bone phase scintigraphic images of the axial skeleton. (**A**) Left lateral view of the cranial cervical vertebrae. (**B**) Left lateral view of the caudal cervical vertebrae. (**C**) Lateral view of the left scapula and withers. (**D**) Left dorso-45–lateral oblique view of the thoracic vertebrae. (**E**) Left dorso-45–lateral oblique view of the thoracolumbar and lumbar area. (**F**) Left dorso-45–lateral oblique view of the pelvis. (**G**) Dorso-20–caudal oblique view of the pelvis. (**H**) Dorsal view of lumbar area. APJ: articular process joint, SP: spinous process, TS: tuber sacrale, DSIL: dorsal sacro-iliac ligament, SIJ: sacro-iliac joint, TI: tuber ischiaticum.

**Figure A2:**
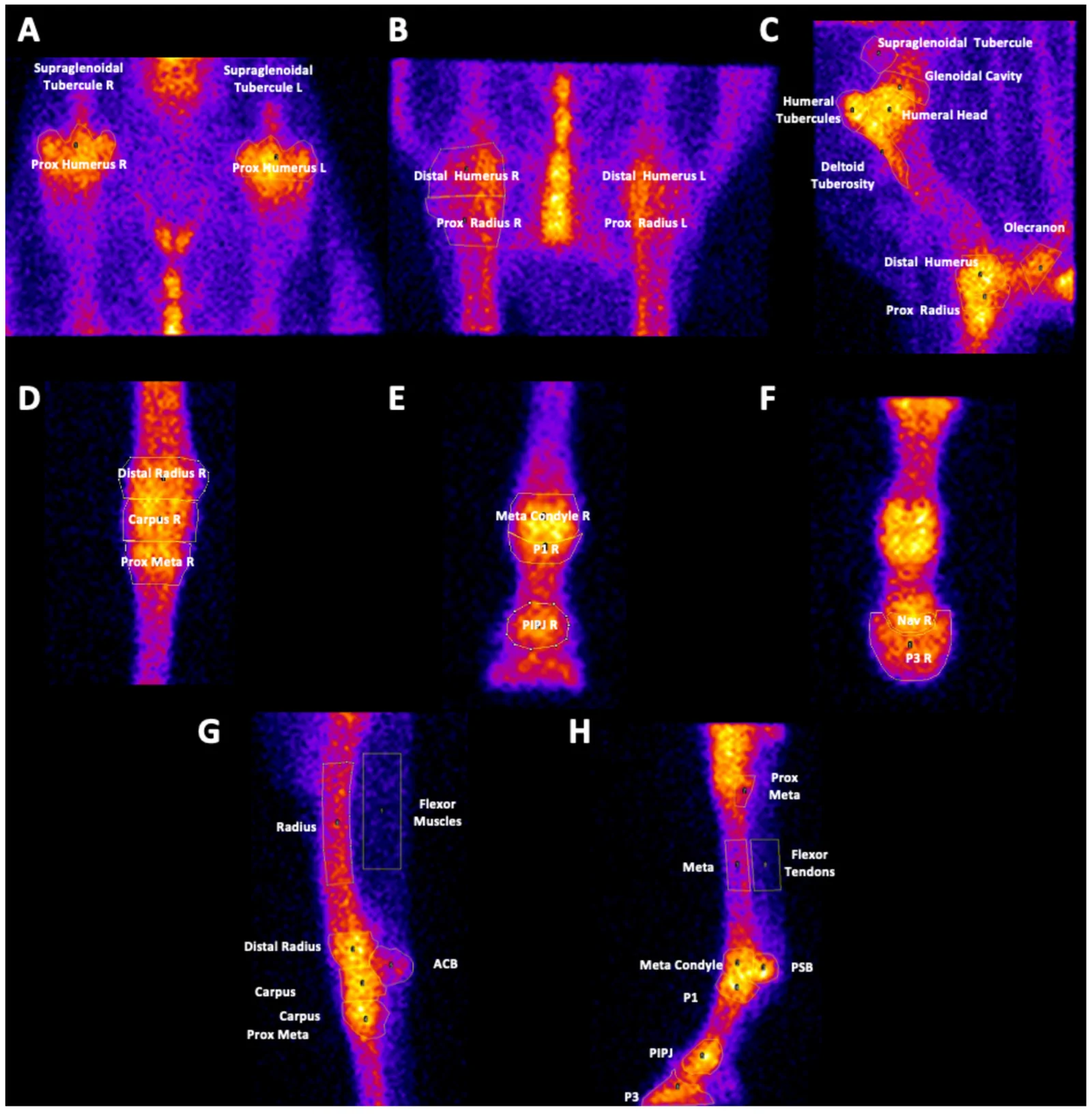
Bone phase scintigraphic images of the front limbs. (**A**) Cranial-30–distal oblique view of the humeri. (**B**) Cranial view of the elbows. (**C**) Latreal view of the left humerus, elbow and shoulder. (**D**) Dorsal view of the carpi. (**E**) Dorsal view of the metacarpal and digital area. (**F**) Solar view of the left front foot. (**G**) Lateral view of the left carpus. (**H**) Lateral view of the metacarpal and digital area of the left frontlimb. PIPJ: proximal interphalangeal joint, Nav: navicular bone, ACB: accessory carpal bone, PSB: proximal sesamoid bone.

**Figure A3:**
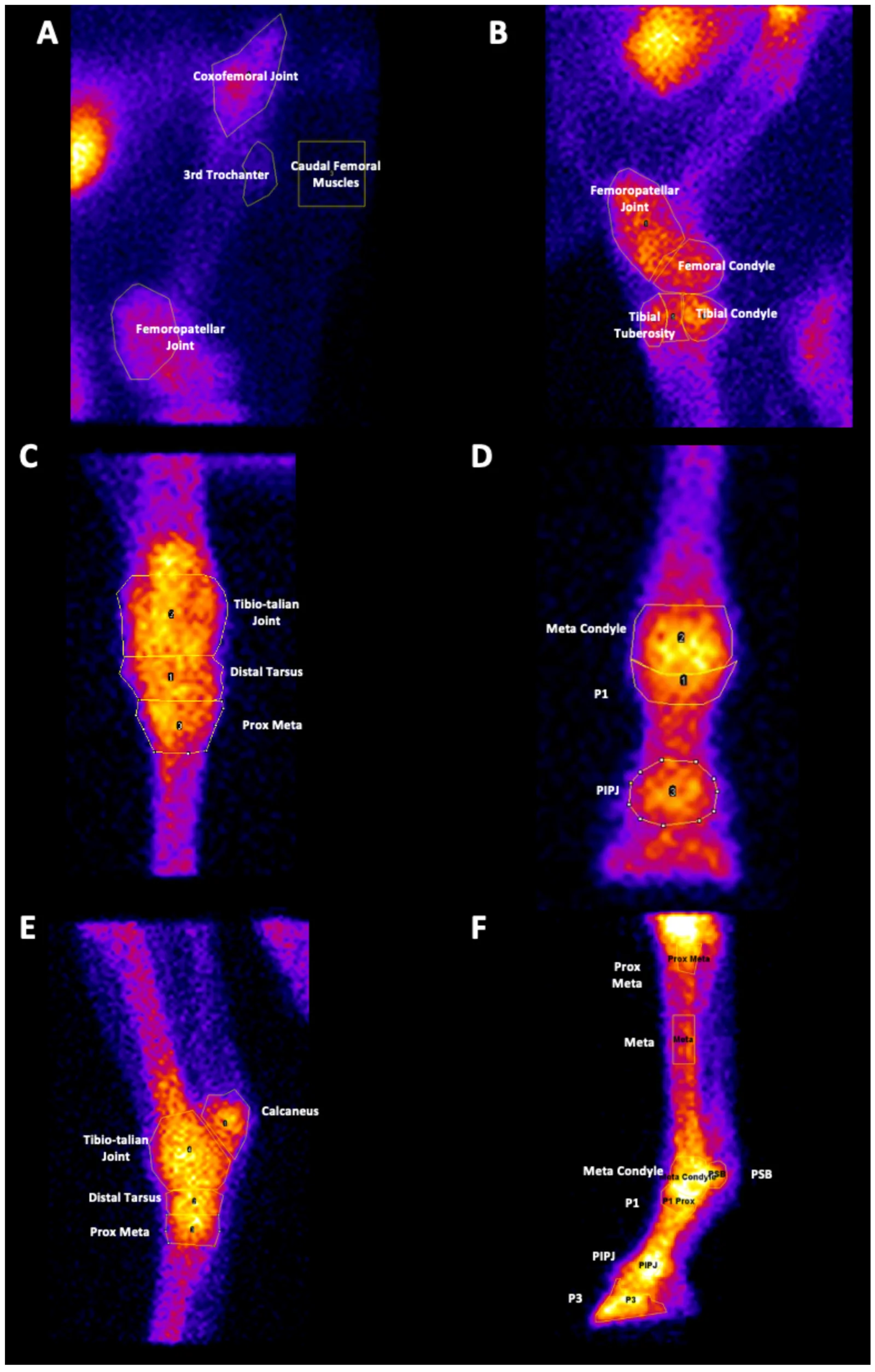
Bone phase scintigraphic images of the hind limbs. (**A**) Lateral-30–cranial oblique view of the left femur. (**B**) Lateral view of the left stifle. (**C**) Plantar view of the tarsi. (**D**) Lateral view of the left tarsus. (**E**) Plantar view of the metatarsal and digital area. (**F**) Lateral view of the metatarsal and digital area of left hindlimb. PIPJ: proximal interphalangeal joint, PSB: proximal sesamoid bone.

Horses received an intravenous injection of 1 GBq per 100 kg of 99m technetium-dicarboxypropane diphosphonate (99m Tc-DPD; CisBio International, Saclay, France) just after a 30 min training session. Scintigraphic images were acquired with a γ-camera (Millenium MPR; GE Healthcare, Buc, France) equipped with a low-energy, high-resolution collimator three hours after the radiopharmaceutical injection. The rectangular field of view was 520 × 370 mm. Dynamic acquisitions of 30 images of 2 s using a 128 × 128 matrix size were obtained for each view under sedation with 10 to 15 µg/kg detomidine and 40 mg of morphine chlorhydrate. A home-made algorithm was used for movement correction [38] to provide one static image for each acquisition. In total 40 views were acquired and stored in DICOM format for quantitative analysis. Details are provided in Figure A1, Figure A2 and Figure A3.

### 2.4. Image Analysis

Images were analyzed with Fiji software (version 1.48v) [39]. The images were first expanded to a 512 × 512 matrix based on least-squares approximation using the resize plugin for Fiji [40]. The greyscale images were colored by applying the lookup table FIRE and brightness and contrast were automatically adjusted using the built-in tools of Fiji. Right lateral views were horizontally flipped to resemble the contralateral views with dorsal or cranial to the left. Each ROI was manually drawn using a stylus on a drawing tablet and stored. For each horse, all the ROIs were defined on the images from one randomly chosen examination and reported on the image from the other examination. ROIs were repositioned by translation when necessary so that the signal intensity was measured in the exact same ROI for the 2 scintigraphic exams. To quantify radiopharmaceutical uptake (RPU), mean pixel intensity (counts/pixel) was measured in 205 anatomically defined regions of interest (ROIs) described in Appendix A. To assess the repeatability of the measurements, the quantitative analysis was performed twice for 111 ROIs at 2-week intervals for the first 6 examinations.

### 2.5. Data Processing

A dataset was created gathering every ROI of every horse the RPU at week 4 and week 12 along with information on the horse (*age*, *sex*, and *clinical group*), on the examination (*radiopharmaceutical dose*: radiopharmaceutical dose per kg, *sedation dose*: detomidine dose per kg) and the *time to acquisition*, defined as the delay between the start of the acquisition of the first image and the image of the ROI. Z-score normalization of RPU was performed using the mean and standard deviation of the RPU of all the ROIs of the same scintigraphic examination. Signal variation during the protocol was calculated on the normalized values as the difference between the value at week 12 and at week 4 (Z-score difference).

### 2.6. Statistical Analysis

Repeatability was assessed using Bland–Altman plots and Lin’s concordance coefficients for each ROI [41]. A value above 0.7 for Lin’s coefficient was considered satisfactory, and a value above 0.8 indicated a good repeatability, following the previously described classification [42].

A linear mixed-effects model was used to assess the changes in signal capture within each ROI using the normalized RPU as a dependent variable. The analyses were primarily exploratory and aimed at identifying general spatial patterns of scintigraphic variation. To account for the large variability in the dataset, multiple variables were considered to be included as fixed effects, and for an exploratory purpose, a stepwise backward selection was performed for each model based on the Akaike information criterion (AIC) as an estimator of prediction error. Tested fixed effects included the *week* (week 4 or week 12), *age*, *sex*, *radiopharmaceutical dose*, *sedation dose* and *time to acquisition*. The horse was always introduced as a random effect to account for repeated measurements. Models’ assumptions were verified through visual inspection of residual plots: normality was assessed using Q-Q plots, and homoscedasticity was verified through standardized residual analysis. The *p*-value for each fixed effect was obtained using an F-test based on Satterthwaite’s approximation to degrees of freedom. Because a large number of ROIs were analyzed, the results should be interpreted with caution as no formal correction for multiple testing was applied.

To represent the individual differences in bone scintigraphic changes, the pattern of scintigraphic variation in the different ROIs was visualized using a heatmap of the Z-score differences with hierarchical clustering of ROIs and horses. Only ROIs with a minimal median variation in 0.1 Z-score were included in the heatmap.

To evaluate the effect of the presence of osteoarticular lesions on the RPU variation in the axial skeleton, another linear mixed-effects model was fitted using the Z-score difference as the dependent variable. A stepwise backward selection of the fixed and random effects based on the AIC was performed. The initial model included horse and ROI as random effects and group, age, lesion, sex and lesion–sex interaction as fixed effects. As the homoscedasticity assumption was not met for the final model, and as the estimated random effects were not normally distributed suggesting potential outliers, the model was fitted again using the Robust Scoring Equations estimator [43]. The 95% confidence intervals for the fixed effects estimators were obtained by a non-parametric bootstrap method using 2000 iterations of resampling.

The same statistical framework was applied to the dataset restricted to the thoracolumbar group.

All data processing and statistical analysis was performed in R (version 4.5.1) [44] using RStudio software (version 2025.9.1.401) [45] including lmerTest [46], robustlmm [43], ComplexHeatmap [47] and ggplot2 [48] packages. The threshold for statistical significance was set at *p* < 0.05.

## 3. Results

Repeatability was good for most ROIs with a median Lin coefficient of 0.95 (Interquartile Range [0.83; 0,98]) and a mean variation between measures of 2.88% (SD 3.09%). Twelve ROIs (12/111) had a Lin’s coefficient between 0.6 and 0.7 and 10 (10/111) between 0.7 and 0.8.

The variation in normalized RPU (Z-score difference) between the 4th and 12th weeks was very small on average (mean variation < 0.001), but marked differences were observed between ROIs (SD = 0.09, range [−0.28; 0.24]). All ROIs belonging to the first decile of the Z-score differences (i.e., 25 ROIs with the highest RPU decrease) were located on the axial skeleton. Conversely, all ROIs from the 9th decile (largest RPU increase), with the exception of the left temporo-mandibular joint and the right first costovertebral joint, were located in the limbs.

**Figure 1:**
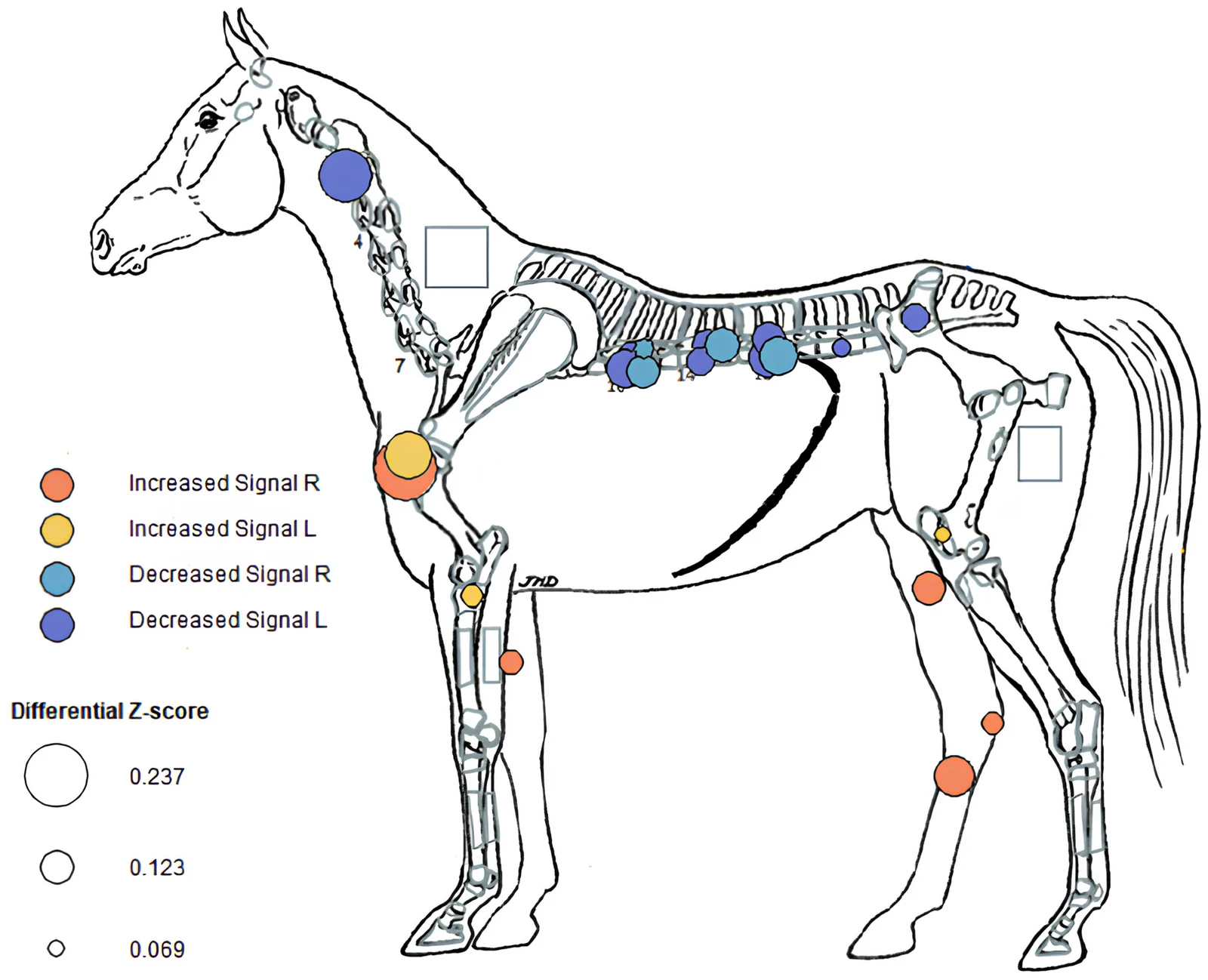
Anatomical localization of regions of interest showing significant normalized radiopharmaceutical uptake variation (differential Z-score) between weeks 4 (before swimming protocol) and week 12 (after swimming protocol). Grey shapes represent localization of region of interest (ROI). Circle color indicates the direction and laterality of the change in radiopharmaceutical uptake: yellow and orange represent increased uptake (right and left views, respectively), whereas dark blue and light blue represent decreased uptake (right and left views, respectively). Circle size is proportional to the magnitude of the differential Z-score (n = 18 horses, 205 ROIs). The threshold for statistical significance was set at *p* < 0.05.

The effect of the week demonstrated a *p*-value inferior to 0.05 in 21 ROIs presented in Table 3 and Figure 1. A RPU decrease was observed in 13 of the ROIs located in the axial skeleton mainly involving the vertebral bodies and articular process joints of the middle thoracic, caudal thoracic, and thoracolumbar area. Most of these findings were consistent when considering the views from the left and from the right side. Eight of the 21 ROIs showed an increased RPU, and were all located on the limbs. The humeral tubercles had an increased RPU both on the right and left lateral views.

**Figure 2:**
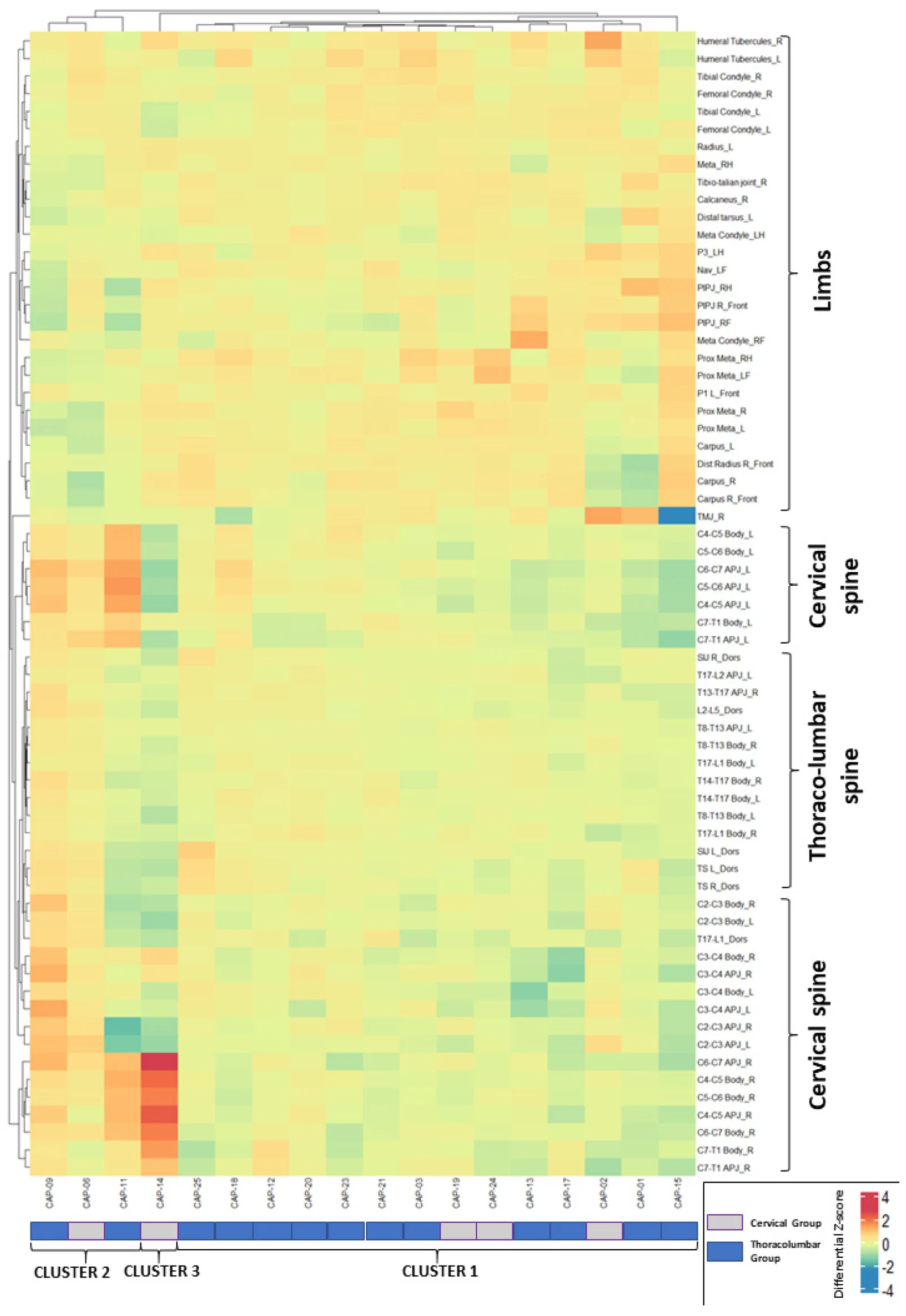
Heatmap of radiopharmaceutical uptake variation (differential Z-score) between week 4 (before swimming protocol) and week 12 (after swimming protocol) across regions of interest (ROIs) and horses (n = 18 horses). Only ROIs with a median absolute differential Z-score greater than 0.1 were included (65 ROIs). Rows represent ROIs and columns represent horses. Both ROIs and horses were hierarchically clustered based on the similarity of their scintigraphic variation patterns. The color scale indicates the magnitude and direction of the differential Z-score: warm colors (yellow to red) indicate an increase in radiopharmaceutical uptake, whereas cool colors (green to blue) indicate a decrease. ROI: region of interest, TL: thoracolumbar, VB: vertebral body, SIJ: sacroiliac joint, APJ: articular process joint, R: right, L: left, RH: right hind, RF: right front, LH: left hind, LF: left front, P1: proximal phalan.

The variation patterns of every horse were evaluated with a heatmap (Figure 2). ROIs clustered by anatomical region. Clusters with low interindividual differences in variation (thoracic and lumbar ROIs) could be distinguished from clusters with very high interindividual variability (caudal neck ROI). Horses could be separated into three hierarchical clusters highlighting various individual changes in different anatomical areas especially in the caudal neck. The largest cluster (n = 14 horses) showed a decreased RPU in all axial ROIs in most horses with increased RPU limited to ROIs in the limbs. A second cluster (n = 3 horses) showed a high RPU increase in caudal and middle cervical areas as well as a moderate increase RPU in the thoracolumbar spine. The last cluster was composed of one single horse showing a highly increased RPU in caudal and middle neck ROIs of the right views and a large decreased RPU in other spinal ROIs. No clear correlation was observed between the clinical groups defined at inclusion and horse clusters based on scintigraphic variation patterns.

**Figure 3:**
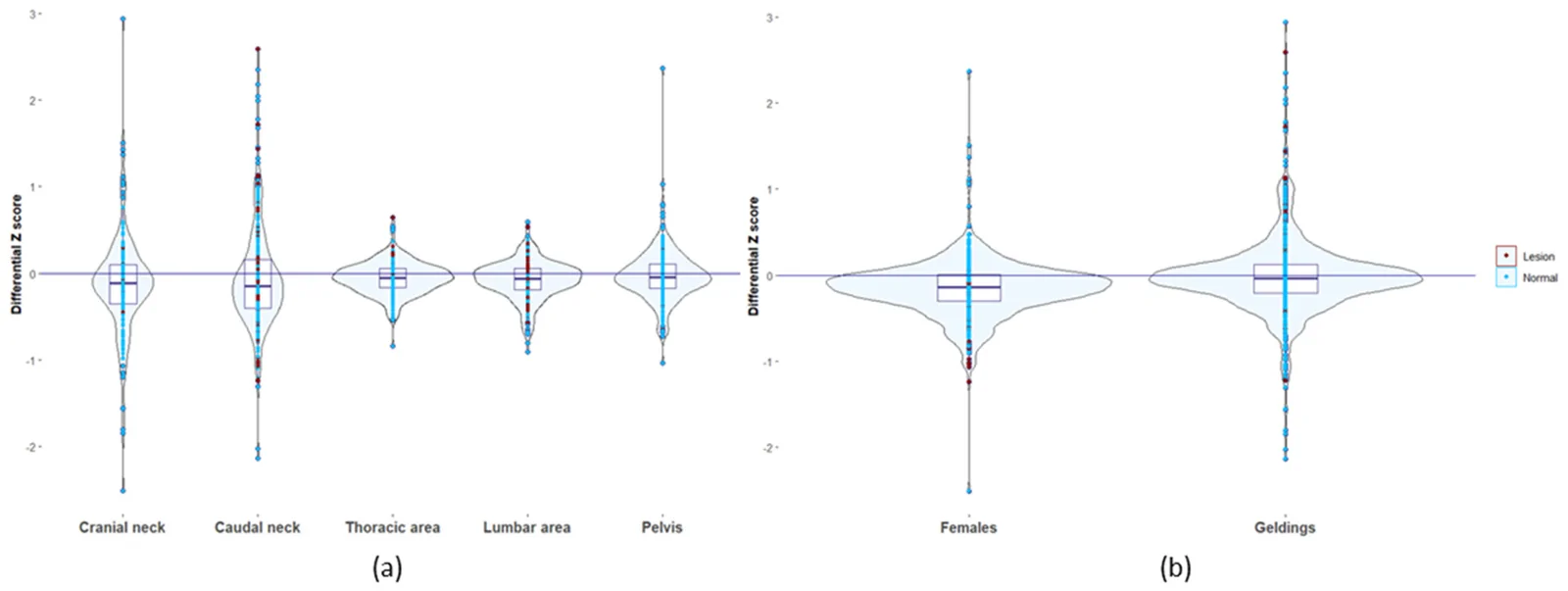
Distribution of radiopharmaceutical uptake variation (differential Z-score) between weeks 4 and 12 in axial skeletal regions of interest (ROIs). Each point represents the differential Z-score calculated for one ROI. Points are colored according to the presence or absence of lesions in the corresponding ROIs. Multiple ROIs were obtained from each horse (n = 18 horses, 83 ROIs). Violin plots represent the distribution of the data, with embedded boxplots indicating the median and interquartile range. The horizontal red line indicates no change in Z-score. (**a**) Distribution of differential Z-scores according to anatomical region. (**b**) Distribution of differential Z-scores according to sex.

To explore potential factors associated with RPU variation in the axial skeleton, an additional linear mixed-effects model was fitted. Axial skeleton ROIs (83 ROIs) were separately evaluated to identify potential factors affecting scintigraphic signal variation, especially the group and the presence of radiographic lesions in the ROIs but also the age and sex of the horse. After a backward stepwise selection process, the linear mixed-effects model included the horse as a random effect and the sex, the presence of a lesion and the interaction terms between sex and lesion as fixed effects. Estimates and confidence intervals are shown in Table 4. The model demonstrated a significant effect of the sex of the horse and the presence of a radiographic lesion on the RPU variation, also with a significant interaction between those terms. Females were more likely to show a decreased RPU. If a lesion was observed, the RPU was more likely to decrease, and this effect was stronger in females than geldings. Boxplots of differential Z-scores in different anatomical area and in females and geldings are presented in Figure 3.

The same analysis was repeated in the thoracolumbar group only (n = 13 horses) and yield very similar results (Supplementary Tables S1 and S2). All the 21 ROIs previously significant remained significant (n = 18 horses, 205 ROIs). Three additional ROIs reached significance (right femoro-patellar joint, left dorsal sacroiliac ligament and left caudal thoracic vertebral body). When evaluating the factors influencing the variation in RPU, the sex, the presence of a lesion and the interaction between the two remained significant with similar estimates (n = 13 horses, 83 ROIs).

## 4. Discussion

### 4.1. Tolerance of Swimming in Horses with Axial Lesions

After a 2-month cross-training program with combined swimming and terrestrial work, this exploratory study did not reveal any significant scintigraphic worsening of the axial lesions. This finding is consistent with previously published data on the same group of horses showing no significant variation in the back movements during trot in straight line [37]. Those combined findings support the good tolerance of swimming in horses with axial lesions even when associated with clinical manifestations. Swimming should not be considered as contraindicated in the rehabilitation program of such horses, but its use should be closely monitored to ensure good clinical tolerance allowing physical conditioning while reducing the load on the skeleton on a case-by-case basis.

Until now, experts’ recommendations have restricted the use of swimming to horses free of thoracolumbar pain [14,49]. Such recommendation was based on the alleged extension of the cervical, thoracolumbar and lumbosacral area of swimming horses. However, recent kinematic studies challenge this concept by demonstrating considerable interindividual variation in swimming technique and in neck and back posture during swimming [9,11]. Our findings support this more nuanced interpretation and suggest that spinal posture alone may not accurately reflect mechanical loading of the axial skeleton.

The apparent discrepancy between spinal extension during swimming and the absence of scintigraphic worsening may be explained by the unique biomechanics of aquatic exercise. Biomechanical stresses imposed on the axial skeleton during complete immersion are greatly different compared to terrestrial work. According to the bow-and-string model of the equine back, the vertebral column is subjected under normal conditions to ventral compressive forces and dorsal tensile forces. The mechanical load applied to the spine is determined by active abdominal muscle contraction which promotes flexion of the back and by the weight of the abdominal viscera which promotes extension of the back [50]. One possible explanation for the apparent tolerance of swimming is that the buoyancy property of water counteracts the vertical load imposed by the weight during swimming [49], thereby reducing the passive extension load applied to the axial skeleton. Swimming is also associated with activation of several muscle groups, including neck extensors such as the splenius [51]. The extended position of the axial skeleton observed in some swimming horses may therefore result more from active muscle contraction more than passive loading, which may contribute to improved tolerance of this posture.

### 4.2. Possible Mechanisms Underlying Reduced Vertebral Radiopharmaceutical Uptake

One surprising finding of this study was the overall tendency toward reduced radiopharmaceutical uptake within the axial skeleton, particularly in regions with pre-existing radiographic abnormalities. Although this exploratory study was not designed to demonstrate definitive treatment effects, this pattern may indicate reduced bone remodeling and osteoblastic activity associated with lower mechanical stress.

Rehabilitation for managing neck and back pain or dysfunction relies on control exercise training with the aim of strengthening and improving motor control of hypoaxial and epaxial muscles. Several aspects of the training program of this study worked toward the same aim. Incorporating a swimming session into a show-jumping training program may have contributed to reach such goals by recruiting abdominal and iliopsoas muscles during hindlimb protraction and engaging gluteal and caudal femoral muscles during propulsion. However, many other factors in the training program are likely to have contributed to this effect on bone stress, especially the change in training conditions and riders.

### 4.3. Interindividual Variability

Despite the overall trend, marked individual variability was observed, particularly in the caudal cervical region. No correlation was observed between variation patterns and horses’ clinical group or age. Sex appeared to influence scintigraphic variation; however, this should be interpreted with caution given the small number of mares included in the study (6/18) and therefore requires further investigation. Additional confounding factors may have contributed to this observation. For example, some horses, all mares, showed marked balky behavior at inclusion that improved during the program. The low number of horses in the cervical group did not allow a group effect to be clearly demonstrated, but those horses contributed to increasing the variability. It may also have worked as a cofounding factor for the sex effect as only one mare (1/5) was present in the group compared to five (5/13) in the thoracolumbar group. Other factors could have contributed to this variability and should be investigated. Swimming technique and especially neck position could have influenced the variation in radiopharmaceutical uptake and should be considered in further studies.

### 4.4. Effect on the Appendicular Skeleton

Unlike the axial skeleton, the appendicular skeleton showed only limited changes. The most consistent adaptation was an increase in radiopharmaceutical uptake in the humeral tubercles. Other scintigraphic changes were inconsistently observed across the limbs and were of small magnitude. The exploratory design of this study calls for increased caution in the interpretation of such individual findings and does not confirm any causal relationship. Forelimb protraction underwater requires increased muscular effort to overcome water resistance [49]. During swimming, the shoulder and elbow joints, as well as other joints, exhibit a greater range of motion with increased maximal flexion [10,13] and their angular velocity is reduced during protraction compared with retraction [13]. These kinematic features are associated with increased muscle activation, as electromyographic studies have demonstrated greater activation of the brachiocephalicus and triceps brachii during swimming [51]. Although activation of the biceps brachii during swimming has not been evaluated in horses, it is possible that mechanical stresses on the bicipital apparatus increase during swimming, potentially leading to bone adaptation.

### 4.5. Interest of Quantitative Scintigraphy for Bone Metabolism Follow-Up

Scintigraphy was used in this study to document the evolution of bone osteoblastic metabolism. This imaging technique has been used to diagnose various axial musculoskeletal injuries [26,28,29,30,31,52] and to monitor the response to medical or rehabilitation treatment [23,24]. Radiopharmaceutical uptake has been shown to correlate well with clinical manifestations of back pain in horses with facet joints arthropathy [26] and with abnormalities of the interspinous spaces in horses with kissing spines [27]. Scintigraphy has been shown to have a high negative predictive value, meaning that low radiopharmaceutical uptake is a good predictor of the absence of clinically significant lesions [26]. These findings support the use of scintigraphy for monitoring the clinical relevance of axial skeletal lesions. The ability to perform objective quantitative measurements is particularly valuable for research purposes. Multiple methods of quantitative and semi-quantitative analysis have been developed for specific areas such as the spinous processes [27,53,54,55], facet joints [25,38], sacroiliac joints [56,57], fatigue fractures [58], feet [59] and distal limbs [60,61]. To our knowledge, this study is the first to describe a quantitative approach for analyzing the whole-body scintigraphic profile. The method proved to be repeatable and reliable and does not rely on the use of one specific reference region. It therefore enables longitudinal scintigraphic follow-up in exploratory prospective studies.

### 4.6. Study Limitations

This study has several limitations. Considering the number of statistical tests, the results should be interpreted with caution. As the study is exploratory, statistical tests were used as a tool to identify most relevant region of RPU changes but not as definitive proof of significance. Additionally, to overcome the inherent variability of RPU in scintigraphic examinations, several possible exploratory variables were tested for each model, and a stepwise backward selection method based on AIC was used. This method may have increased the risk of overfitting the data and could have led to model instability and biased parameter estimation. The choice of this method was motivated by the fact that the influence of cofounding factors may be different for each ROI. For example, *time to acquisition* would have greater interindividual variability and therefore greater influence in the model for images acquired at the end of the examination. A backward selection offered the ability to select a specific set of dependent variables for each ROI in a standardized and objective manner.

The relatively low statistical power has limited the ability of the study to detect small changes. In particular, the small number of horses in the cervical group did not allow for a direct comparison to be made between the two groups. Further studies are therefore necessary to explore the consequences of swimming in horses with severe cervical lesions.

Due to the absence of a control group, this exploratory study cannot establish causality or treatment efficacy, but it provides objective evidence that aquatic exercise was not associated with worsening of bone metabolic activity and may contribute to favorable skeletal adaptation. Larger controlled studies are now required to determine the respective contributions of swimming and terrestrial rehabilitation.

## 5. Conclusions

Overall, the results of this exploratory study indicate that a cross-training program including swimming was not associated with increased RPU in the axial skeleton of horses with neck and back pain. A mild tendency toward reduced radiopharmaceutical uptake in the axial skeleton was observed despite marked interindividual variability in scintigraphic changes. The conclusions drawn here are constrained by the study limitations related to the exploratory nature of the design and the relatively low statistical power. Further controlled studies are needed to better characterize the effects of swimming and to determine the factors influencing these variations. In particular, a controlled study including a large cohort of horses with mainly cervical lesions is required to clarify the effect of swimming in this population.

## Figures and Tables

**Table 1:** Characteristics of the horses included in the study and description of axial skeletal lesions.

Horse	Sex	Age (y)	Height (cm)	Body Mass (kg)	Lesions (Location)	Clinical Group
01	Mare	11	164	504	DSP impingement (T13–T14, T16–T17), OA (T15–T16, T18-L3), Right SIJA, Right and left LSITJA Vertebral symphysis abnormality (C6–C7, C7–T1)	Thoracolumbar Group
02	Mare	14	160	502	OA (C5–T1), DSP impingement (T13–T16)	Cervical Group
03	Gelding	12	148	424	OA (T15–T16, T18–L1 > L1–L4), DSP impingement (T15–T18) Right SIJA, Right and left LSITJA Vertebral symphysis abnormality (C6–C7, C7–T1) External occipital crest remodeling	Thoracolumbar Group
06	Gelding	10	166	566	Vertebral symphysis abnormality (C6–C7), OA (C6–T1), R1 hypogenesia, OA (T17–L3), Thoracic lordosis, DSP impingement (T17–T18) Right and left SIJA, External occipital crest remodeling	Cervical Group
09	Gelding	11	170	618	Vertebral body dysplasia (T9–T10 cuneiformis vertebrae), Thoracic lordosis, DSP impingement (T12–T16) Vertebral symphysis abnormality (L6–S1) Right and left SIJA, OA (L2–L3), Vertebral symphysis abnormality (C6–T1), External occipital crest remodeling	Thoracolumbar Group
11	Gelding	13	166	594	DSP impingement (T13–T17), OA (T14–T18) Right and left SIJA, OA (C6–C7), Vertebral symphysis abnormality (C6–C7), External occipital crest remodeling	Thoracolumbar Group
12	Gelding	11	170	520	DSP impingement (T13–T18), OA (T14–T17, T18–L2), Vertebral symphysis abnormality (L6–S1), OA (C6–T1), Vertebral symphysis abnormality (C6–C7),	Thoracolumbar Group
13	Gelding	10	177	562	OA (L1–L3) Left LSITJA, Right SIJA, OA (C6–T1), External occipital crest remodeling	Thoracolumbar Group
14	Gelding	9	177	596	OA (C6–C7), Vertebral symphysis abnormality (C6–T1), Vertebral symphysis abnormality (L6–S1) Right SIJA, OA (T14–T16), DSP impingement (T12–T15), External occipital crest remodeling	Cervical Group
15	Mare	8	166	560	OA (T18–L2), Lumbar kyphosis and scoliosis, DSP impingement (T12–T18), OA (C5–T1)	Thoracolumbar Group
17	Gelding	9	180	616	Vertebral symphysis abnormality (L6–S1) OA (T18–L2), Thoracic lordosis, DSP impingement (T15–T16, L4–L5) OA (C6–T1), Vertebral symphysis abnormality (C6–C7), External occipital crest remodeling	Thoracolumbar Group
18	Gelding	5	167	580	Thoracolumbar lordo-kyphosis and lumbar scoliosis, OA (T15–T18)	Thoracolumbar Group
19	Gelding	5	163	528	OA (C6–C7), Vertebral symphysis abnormality (C6–C7), Vertebral symphysis abnormality (L6–S1), Left SIJA, OA (L2–L4), DSP impingement (L4–L6)	Cervical Group
20	Gelding	5	167	550	OA (T15–L2), DSP impingement (T18–L1), Vertebral symphysis abnormality (L6–S1), Right and left LSITJA, Left SIJA,	Thoracolumbar Group
21	Mare	8	166	524	Ventral spondylosis (T12–T13), DSP impingement (T14–L2), OA (T15–T16) Vertebral symphysis abnormality (L6–S1), Right and left LSITJA, Right SIJA, OA (C6–C7), Left CVJA 1	Thoracolumbar Group
23	Mare	9	168	564	Ventral spondylosis (T11–T12) OA (T18–L3), Vertebral symphysis abnormality (L6–S1), Vertebral symphysis abnormality (C6–C7), Right SIJA, OA (C6–T1), Left CVJA 1	Thoracolumbar Group
24	Gelding	7	164	519	OA (C5-T1), Vertebral symphysis abnormality (C6–T1 and C3–C4), DSP impingement (T15–T17), Vertebral symphysis abnormality (L6–S1), Right and left SIJA	Cervical Group
25	Mare	10	158	494	Vertebral symphysis abnormality (L6–S1), Right and left SIJA, DSP impingement (T12–T17) Vertebral body dysplasia (C6) OA (T18–L3), CVJA 1 External occipital crest remodeling	Thoracolumbar Group

OA: osteoarthritis of the articular processes-synovial intervertebral joints; DSP: dorsal spinal process; SIJA: sacroiliac joint arthropathy; LSITJA: lumbo-sacral intertransverse joint arthropathy; CVJA: costovertebral joint arthropathy; C: cervical vertebra; T: thoracic vertebra; R: rib; L: lumbar vertebra.

**Table 2:** Weekly training schedule and swimming workload followed by the horses during the land-training and cross-training program.

Day	Phase 1—Land Training	Phase 2—Cross-Training								
		All Weeks	W5	W6	W7	W8	W9	W10	W11	W12
**Monday**	Walker (40 min) + flatwork (50 min)	Walker (40 min)	240 m	440 m	605 m	605 m	660 m	770 m	770 m	770 m
**Tuesday**	Walker (40 min) + gymnastic-cavaletti (warming-up + 20–25 cavaletti < 50 cm)									
**Wednesday**	Walker (40 min) + gallop (warming-up + 3 × 3 min)	Walker (40 min)	330 m	440 m	550 m	660 m	605 m	660 m	660 m	605 m
**Thursday**	Walker (40 min) + jumping (warming-up + 20–25 jumps approximately 1 m)									
**Friday**	Walker (40 min) + flatwork (50 min)	Walker (40 min)	330 m	440 m	715 m	770 m	770 m	935 m	935 m	-
**Saturday**	Walker (30 min) + lunge work (25 min)									
**Sunday**	Rest day + walker (30 min)									

**Table 3:** Regions of interest (ROIs) with a significant effect of the week (n = 18 horses, 205 ROIs). The threshold for statistical significance was set at *p* < 0.05. All *p*-value below the threshold are written in bold red.

ROI	Variation	Fixed Effects Coefficient						*p*-Value						
		Week	Time	Age	Sex	Dose	Sedation	Week	Time	Age	Sex	Dose	Sedation	Horse
**TL VB L**	−0.146	−0.146	-	-	-	-	-	**0.003**	0.104	0.094	0.337	0.811	0.983	**<0.001**
**Cranial Thoracic APJ L**	−0.098	−0.098	-	0.047	-	-	-	**0.004**	0.518	**0.001**	0.109	0.364	0.407	**0.008**
**SIJ L**	−0.122	−0.122	-	-	-	-	-	**0.007**	0.081	0.102	0.644	0.302	0.739	**<0.001**
**Cranial Thoracic VB R**	−0.119	−0.119	-	0.050	-	-	-	**0.007**	0.159	**0.021**	0.790	0.867	0.546	**<0.001**
**Cranial Thoracic VB L**	−0.161	−0.143	-	-	-	-	1.668	**0.008**	0.461	0.053	0.842	0.615	**0.019**	**0.001**
**Radius RF**	0.094	0.094	-	-	-	-	-	**0.010**	0.573	0.971	0.271	0.229	0.464	**0.005**
**Humeral tubercles RF**	0.251	0.251	-	-	-	-	-	**0.011**	0.347	0.099	0.135	0.433	0.959	**0.010**
**Tibial condyle RH**	0.138	0.134	-	−0.083	-	−0.206	-	**0.013**	0.051	**0.009**	0.058	**0.022**	0.073	**<0.001**
**TL VB R**	−0.156	−0.156	-	0.059	-	-	-	**0.015**	0.992	**0.015**	0.197	0.413	0.366	**0.019**
**Humeral tubercles LF**	0.189	0.183	-	-	−0.423	−0.291	-	**0.018**	0.399	0.365	**0.031**	**0.011**	0.166	**0.007**
**Proximal metatarsus RH**	0.166	0.197	-	−0.092	-	-	2.963	**0.022**	0.377	**<0.001**	0.124	0.251	**0.001**	0.442
**Caudal Thoracic APJ L**	−0.115	−0.115	-	0.048	-	-	-	**0.023**	0.247	**0.031**	0.791	0.314	0.733	**<0.001**
**Femoro-patellar joint LH**	0.075	0.062	−0.020	−0.083	-	−0.224	1.635	**0.028**	**<0.001**	**0.004**	0.261	**<0.001**	**0.001**	**<0.001**
**P1 dorsal LF**	0.135	0.135	-	-	-	-	-	**0.032**	0.100	0.537	0.873	0.938	0.413	**<0.001**
**TL APJ L**	−0.118	−0.118	-	-	-	-	-	**0.032**	0.167	0.141	0.508	0.444	0.542	**<0.001**
**TL area Dorsal**	−0.206	−0.206	-	-	-	-	-	**0.038**	0.433	0.600	0.509	0.969	0.958	**0.017**
**Calcaneus RH**	0.090	0.090	-	−0.044	-	-	-	**0.040**	0.678	**0.039**	0.087	0.396	0.113	**0.001**
**Caudal Thoracic VB R**	−0.128	−0.128	-	0.049	-	-	-	**0.042**	0.765	**0.043**	0.170	0.489	0.704	**0.024**
**Proximal radius LH**	0.081	0.075	-	-	-	−0.289	-	**0.042**	0.400	0.155	0.335	**0.001**	0.147	**<0.001**
**Femoral condyle RH**	0.116	0.116	-	−0.072	-	-	-	**0.043**	0.254	**0.003**	0.097	0.076	0.070	**0.008**
**Radius LF**	0.078	0.078	-	-	-	-	-	**0.043**	0.499	0.660	0.474	0.129	0.555	**0.019**
**Lumbar area dorsal**	−0.147	−0.126	0.015	-	-	-	-	**0.046**	**0.049**	0.981	0.341	0.768	0.952	**<0.001**
**Tibio-talian joint RH**	0.102	0.122	-	−0.050	-	-	1.898	**0.048**	0.197	**0.042**	0.517	0.502	**0.019**	**0.003**

**Table 4:** Linear mixed-effects model for prediction of signal variation (n = 18 horses, 83 ROIs).

**Random Effects**				
**Variable**	Variance	SD		
**Horse**	0.018	0.13		
**Residuals**	0.083	0.29		
**Fixed Effects**				
**Variable**	Estimate	SD	t-value	Confidence interval
**Intercept** (*Female*, *Lesion*)	−0.27	0.064	−4.30	−0.33; −0.22
**Lesion** (*Normal*)	0.14	0.034	4.22	0.08; 0.21
**Sex** (*Gelding*)	0.25	0.077	3.28	0.19; 0.33
**Sex·Lesion** (*Gelding*, *Normal*)	−0.19	0.041	−4.66	−0.27; −0.11

## Data Availability

Additional data are available upon reasonable request from the corresponding author.

## Supplementary Materials

The following supporting information can be downloaded at: https://www.mdpi.com/article/10.3390/ani16162526/s1, Table S1: Region of interest (ROI) with a significant effect of the week (n = 15 horses, 205 ROI). The threshold for statistical significance was set at *p* < 0.05; Table S2: Linear mixed-effect model for prediction of signal variation (n = 15 horses, 83 ROI).

## Abbreviations

The following abbreviations are used in this manuscript:

ECVSMR	European College of Veterinary Sports Medicine and Rehabilitation
ACVSMR	American College of Veterinary Sports Medicine and Rehabilitation
ECVDI	European College of Veterinary Diagnostic Imaging
ROI	Region of interest
RPU	Radiopharmaceutical uptake
AIC	Akaike information criterion
