# Supplementary material for: Quantitative Bone Scintigraphic Follow-Up After a 2-Month Cross-Training Program Including Swimming in Showjumping Horses with Back and Neck Pain
Source: Animals (Basel). 2026 Aug 13;16(16):2526. doi: 10.3390/ani16162526 (PMC13508862; doi:10.3390/ani16162526)
Supplement: Supplementary file 1 [file animals-16-02526-s001.zip › animals-4444780-supplementary.pdf]

**Supplementary Table S1.** Region of interest (ROI) with a significant effect of the week (n=15 horses, 205 ROI). The threshold for statistical significance was set at p<0.05.

| ROI | VARIATION | Fixed Effect Coefficient | P-VALUES | | | | | | |
|---|---|---|---|---|---|---|---|---|---|
| | | Week | Week | Time | Age | Sex | Dose | Sedation | Horse |
| TL VB L | -0.151 | -0.151 | **0.002** | 0.081 | 0.106 | 0.307 | 0.744 | 0.929 | **<0.001** |
| Cranial Thoracic APJ L | -0.101 | -0.101 | **0.003** | 0.570 | **<0.001** | 0.079 | 0.273 | 0.263 | **0.005** |
| Cranial Thoracic VB R | -0.124 | -0.124 | **0.005** | 0.160 | **0.014** | 0.746 | 0.769 | 0.591 | **<0.001** |
| SIJ L | -0.127 | -0.127 | **0.005** | 0.078 | 0.078 | 0.685 | 0.260 | 0.793 | **<0.001** |
| Tibial condyle RH | 0.138 | 0.149 | **0.005** | 0.060 | **0.002** | **0.045** | **0.015** | **0.043** | **<0.001** |
| Cranial Thoracic VB L | -0.167 | -0.151 | **0.006** | 0.453 | **0.040** | 0.808 | 0.656 | **0.028** | **0.001** |
| Humeral tubercles RF | 0.256 | 0.256 | **0.011** | 0.343 | 0.109 | 0.132 | 0.437 | 0.971 | **0.010** |
| Radius RF | 0.097 | 0.097 | **0.013** | 0.517 | 0.788 | 0.328 | 0.327 | 0.606 | **0.006** |
| TL VB R | -0.162 | -0.162 | **0.014** | 0.992 | **0.011** | 0.177 | 0.365 | 0.435 | **0.021** |
| Humeral tubercles LF | 0.193 | 0.187 | **0.018** | 0.398 | 0.390 | **0.030** | **0.012** | 0.161 | **0.006** |
| Caudal Thoracic APJ L | -0.120 | -0.120 | **0.020** | 0.259 | **0.025** | 0.762 | 0.281 | 0.665 | **<0.001** |
| Proximal metatarsus RH | 0.168 | 0.200 | **0.025** | 0.385 | **<0.001** | 0.139 | 0.234 | **0.002** | 0.464 |
| TL APJ L | -0.123 | -0.123 | **0.031** | 0.177 | 0.119 | 0.484 | 0.411 | 0.508 | **<0.001** |
| Femoro-patellar joint LH | 0.062 | 0.059 | **0.033** | **<0.001** | **0.004** | 0.084 | **0.001** | **<0.001** | **<0.001** |
| Caudal Thoracic VB R | -0.133 | -0.133 | **0.036** | 0.783 | **0.033** | 0.155 | 0.446 | 0.754 | **0.022** |
| TL area Dorsal | -0.216 | -0.216 | **0.037** | 0.438 | 0.594 | 0.494 | 0.966 | 0.970 | **0.018** |
| Femoro-patellar joint RH | 0.083 | 0.103 | **0.038** | 0.308 | **0.029** | 0.121 | 0.690 | **0.010** | **<0.001** |
| P1 dorsal LF | 0.134 | 0.134 | **0.040** | 0.110 | 0.556 | 0.855 | 0.922 | 0.436 | **<0.001** |
| Lumbar area dorsal | -0.154 | -0.132 | **0.041** | **0.047** | 0.932 | 0.339 | 0.816 | 0.985 | **<0.001** |
| DSIL L | -0.077 | -0.077 | **0.043** | 0.522 | 0.942 | 0.997 | 0.602 | 0.121 | **<0.001** |
| Proximal radius LH | 0.080 | 0.074 | **0.044** | 0.369 | 0.167 | 0.343 | **<0.001** | 0.153 | **<0.001** |
| Femoral condyle RH | 0.116 | 0.116 | **0.048** | 0.254 | **0.003** | 0.097 | 0.083 | 0.069 | **0.009** |
| Caudal Thoracic VB L | -0.107 | -0.107 | **0.049** | 0.688 | 0.073 | 0.367 | 0.724 | 0.156 | **<0.001** |
| Radius LF | 0.079 | 0.079 | **0.049** | 0.660 | 0.883 | 0.506 | 0.178 | 0.434 | **0.021** |

**Supplementary Table S2.** Linear mixed-effect model for prediction of signal variation (n=15 horses, 83 ROI).

| Random Effects | | | | |
|---|---|---|---|---|
| **Variable** | Variance | SD | | |
| **Horse** | 0.029 | 0.17 | | |
| **Residuals** | 0.13 | 0.36 | | |
| **Fixed Effects** | | | | |
| **Variable** | Estimate | SD | t-value | Confidence interval |
| **Intercept** *(Female, Lesion)* | -0.32 | 0.097 | -3.32 | -0.51; -0.14 |
| **Lesion** *(Normal)* | 0.16 | 0.052 | 3.04 | 0.06; 0.26 |
| **Sex** *(Gelding)* | 0.34 | 0.116 | 2.9 | 0.11; 0.56 |
| **Sex.Lesion** *(Gelding, Normal)* | -0.20 | 0.062 | -3.18 | -0.32; -0.07 |
